# Accuracy of automated segmentation of gluteus and paraspinal muscles in patients with hip osteoarthritis using TotalSegmentator in 3Dslicer

**DOI:** 10.1186/s42836-026-00412-8

**Published:** 2026-07-08

**Authors:** Hyonmin Choe, Shogo Yokota, Ryohei Iino, Koki Abe, Hironori Yamane, Yuta Hieda, Hiroyuki Ike, Ken Kumagai, Naomi Kobayashi, Yutaka Inaba

**Affiliations:** 1 Department of Orthopaedic Surgery, Yokohama City University, Yokohama, Kanagawa 236-0004 Japan; 2 Yokohama City University School of Medicine, Yokohama, Kanagawa 236-0004 Japan; 3 Department of Orthopaedic Surgery, Yokohama City University Medical Center, Yokohama, Kanagawa 232-0024 Japan

**Keywords:** Muscles, Hip, Artificial intelligence, Segmentation, TotalSegmentator, 3D Slicer

## Abstract

**Background:**

Muscle atrophy is prevalent among patients with hip osteoarthritis, yet quantitative assessment of the peri-hip muscles remains challenging. Although computed tomography (CT) enables objective evaluation, manual segmentation is time-consuming and impractical for routine use. TotalSegmentator, an artificial intelligence-based extension of 3D Slicer, allows rapid automated muscle segmentation. The purpose of this study was to evaluate the accuracy, reproducibility, and clinical relevance of automated peri-hip muscle segmentation using TotalSegmentator in patients with hip osteoarthritis.

**Methods:**

This retrospective cohort study included 53 female patients (106 hips) with bilateral hip osteoarthritis. Automated segmentation using TotalSegmentator was performed for the gluteus maximus, gluteus medius, and paraspinal muscles and compared with manual segmentation. Muscle volume and CT attenuation were analyzed. Agreement and intraobserver reproducibility were assessed using intraclass correlation coefficients (ICCs). Associations with patient demographics, spinopelvic alignment, and hip function, assessed using the modified Harris Hip Score (mHHS), were also examined in exploratory secondary analyses.

**Results:**

TotalSegmentator demonstrated moderate to good reliability for muscle volume and CT attenuation compared with manual segmentation (ICCs 0.47–0.83), with strong correlations between methods (*r* = 0.79–0.96). Muscle volumes measured by TotalSegmentator were significantly smaller, whereas CT attenuation values were significantly higher than those obtained by manual segmentation. Intraobserver reproducibility was excellent, with ICCs of 1.00 for all muscles. Higher CT attenuation demonstrated a weak positive correlation with young age, better sagittal malalignment, and higher postoperative mHHS.

**Conclusion:**

Automated peri-hip muscle segmentation using TotalSegmentator provides rapid, accurate, and reproducible quantitative assessment of muscle volume and quality in patients with hip osteoarthritis.

## Introduction

Osteoarthritis of the hip is a degenerative disease characterized by hip joint deformity that causes hip pain and muscle weakness, leading to a decline in activities of daily living (ADL) and quality of life (QOL). With the aging population, the number of patients affected by this condition is rapidly increasing. Total hip arthroplasty (THA) is a well-established surgical treatment that offers significant improvements in pain relief and hip function, even in advanced stages of osteoarthritis [1]. However, previous studies have demonstrated that patients undergoing THA who present with pronounced preoperative muscle weakness or postural abnormalities are at increased risk of persistent adjacent joint symptoms, including low back and knee pain, and tend to exhibit limited postoperative improvement in gait performance and hip function [2–5]. Therefore, in patients with degenerative conditions such as hip osteoarthritis, comprehensive preoperative assessment of muscle status, together with appropriately tailored rehabilitative interventions, may contribute to improved postoperative outcomes.

Muscle evaluation can generally be categorized into two approaches: direct assessment of muscle strength and imaging-based evaluation using modalities such as magnetic resonance imaging (MRI) and computed tomography (CT). In healthy individuals, direct strength measurement represents a straightforward and practical method. However, in patients with substantial pain related to degenerative conditions such as osteoarthritis, accurate preoperative assessment of true muscle strength is often challenging. Moreover, direct strength measurement involves the activation of multiple muscle groups, making it challenging to isolate and directly assess the targeted muscle. In contrast, imaging modalities such as CT and MRI are well-suited for detailed evaluation of individual muscles. These techniques allow for the segmentation of target muscles from comprehensive imaging datasets, enabling independent assessment of each muscle [6, 7]. In clinical practice, CT scans of the hip region are often obtained preoperatively in patients undergoing THA or with hip fractures. Thus, CT-based muscle analysis represents a feasible alternative for muscle evaluation in these patients [6, 7]. However, conventional CT-based assessment has been limited by the requirement for dedicated software and the substantial time and effort associated with manual segmentation, rendering routine clinical application impractical despite the widespread availability of CT imaging data.

3D Slicer (www.slicer.org) is an open-source software platform freely available to all users [8]. TotalSegmentator is an add-in module that enables automatic segmentation of multiple anatomical structures. By integrating the add-in module TotalSegmentator into 3D Slicer, it becomes possible to automatically segment ten major peri-hip muscles, including the bilateral gluteus muscles (gluteus maximus; G.max, gluteus medius; G.med, and gluteus minimus; G.min), iliopsoas, and paraspinal muscle, from CT or MRI imaging data. This integration enables rapid segmentation that would otherwise require several hours of manual effort, reducing processing time to just a few minutes. The automatically extracted models of peri-hip muscles and skeletal structures can be used not only for volumetric and CT density analysis but also for potential surgical planning applications [9]. However, to date, there have been no studies applying TotalSegmentator in the 3Dslicer technique specifically to patients with hip osteoarthritis. The purpose of this study is to evaluate the accuracy and clinical utility of automated peri-hip muscle segmentation using 3D Slicer and TotalSegmentator from preoperative CT scans in patients with hip osteoarthritis scheduled to undergo THA.

## Patients and methods

### Patient enrollment

**Fig. 1:**
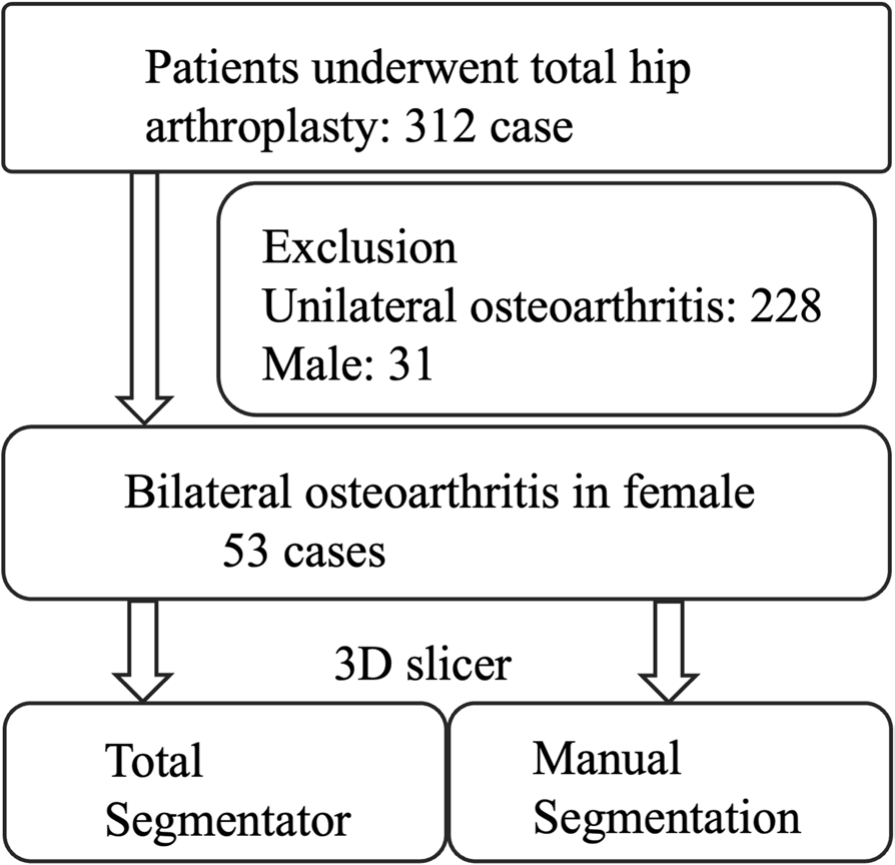
Patient enrollment. A total of 312 patients who underwent total hip arthroplasty were initially screened. Patients with unilateral hip osteoarthritis (*n* = 228) and male patients (*n* = 31) were excluded. The final study cohort consisted of 53 female patients with bilateral hip osteoarthritis. Computed tomography data from the eligible patients were analyzed using two segmentation approaches: automated muscle segmentation with TotalSegmentator implemented in 3D Slicer and conventional manual segmentation for comparison

This retrospective cohort study was approved by our institutional review board (F241000003). Among 312 patients with hip osteoarthritis scheduled to undergo THA at our institution between January 2019 and April 2022, we included 53 female patients diagnosed with bilateral hip osteoarthritis after excluding male patients and those with unilateral hip osteoarthritis (Fig. 1). To specifically evaluate the performance of automated muscle segmentation in the pathological conditions of osteoarthritis, only patients with bilateral hip osteoarthritis were included in this study.

### Accuracy of automated segmentation for muscles

**Fig. 2:**
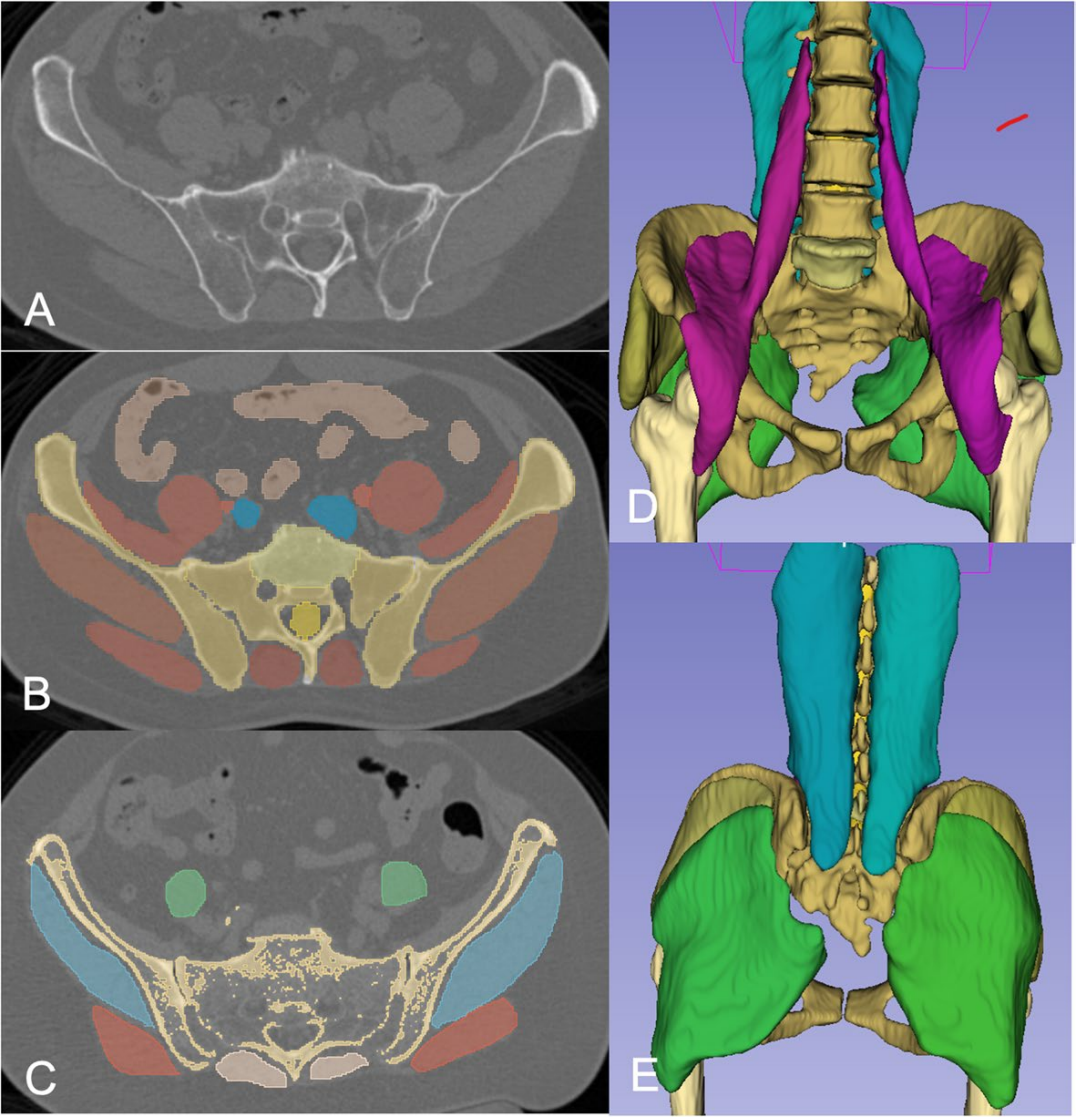
Representative examples of automated and manual peri-hip muscle segmentation. Representative axial computed tomography (CT) images demonstrating peri-hip muscle segmentation using automated TotalSegmentator–based analysis and conventional manual segmentation (**A**–**C**). Segmented muscle groups, including the gluteus maximus, gluteus medius, and paraspinal muscles, were used for quantitative assessment of muscle volume and mean CT attenuation (**D**, **E**)

CT imaging was performed from the T12–L1 intervertebral level to the distal femur using a slice thickness of 1.5 mm. During scanning, a calibration phantom (B-MAS 200; Kyoto-Kagaku, Kyoto, Japan) was placed on the patient’s back to allow for quantitative analysis [6]. Using the TotalSegmentator installed in the open-source software of 3D Slicer [8], the CT datasets of each subject were loaded, and the muscles were segmented slice by slice (Fig. 2A–C) to generate three-dimensional muscle models (Fig. 2D and E). From these models, muscle volume (cm^3^) and CT attenuation values of Hounsfield units (HU) were calculated. Segmentation was performed automatically using TotalSegmentator*,* an AI-based extension of 3D Slicer that enables automated identification of 10 bilateral muscles of G.max, G.med, G.min, Psoas, and paraspinal muscles mentioned above [9, 10]. Paraspinal muscles were defined according to the TotalSegmentator labeling scheme as the autochthonous dorsal musculature, comprising the erector spinae and multifidus muscles. As these muscles are not separately segmented, they were analyzed as a single compartment. For the present study, automated segmentation results for the G.max, G.med, and paraspinal muscles were used for validation and collectively referred to as the TotalSegmentator (TS) group [10, 11]. As a reference for accuracy comparison, manual segmentation of the same muscles was also performed as the manual segmentation (MS) group [6, 7]. Manual segmentation was initially performed by a trained medical student who had received prior instruction in the segmentation protocol. To ensure the accuracy and consistency of the segmentation, all segmented images were subsequently reviewed and verified by a board-certified orthopaedic surgeon. Using a similar segmentation protocol, our previous study reported intra-observer and inter-observer reliability for 3D measurements of the G.med volume at 0.998 (95% CI, 0.996–0.999) and 0.948 (95% CI, 0.887–0.977), respectively [6].

The software used in this study can be downloaded and installed on individual personal computers. Therefore, all image data were managed locally on each user’s computer. CT data were handled in Digital Imaging and Communications in Medicine (DICOM) format. Data handling and privacy protection procedures were conducted in accordance with standard clinical practices for imaging analysis using CT data, ensuring appropriate protection of patient confidentiality.

### Correlation of patients’ demographics with muscle evaluations

We then evaluated the relationships between muscle parameters of muscle volume (cm^3^) and CT attenuation value (HU) obtained using TotalSegmentator and various patient factors, including age, height, weight, body mass index (BMI), preoperative spinopelvic sagittal alignment, and pre- and postoperative hip function. The preoperative spinopelvic parameters—sagittal vertical axis (SVA), lumbar lordosis (LL), and sacral slope (SS)—were measured using the EOS imaging system [12]. Pre- and postoperative hip function was assessed using the Modified Harris Hip Score (mHHS).

### Statistical analysis

To evaluate the reliability between the TS group and MS group, the intraclass correlation coefficient (ICC) was calculated. The correlations of the corresponding measurements between the two groups were further analyzed using Pearson’s correlation coefficient. Differences in the measured values between the TS and MS groups were also assessed using paired t-tests. To examine the intraobserver reliability of muscle segmentation using TotalSegmentator in 3D Slicer, the same CT datasets were re-downloaded and re-analyzed after an interval of one month, and ICCs were calculated. In addition, correlations between patient background factors, preoperative spinopelvic alignment parameters, and postoperative HHS and muscle parameters measured by TotalSegmentator in 3D Slicer were evaluated using Pearson’s correlation coefficient as exploratory secondary analyses. The correlations among the measured muscle parameters were also evaluated using Pearson’s correlation coefficients. All statistical analyses were performed using SPSS software (version 29.0.1; IBM Corp., Armonk, NY, USA), and graphs were generated using GraphPad Prism 8 (GraphPad Software, San Diego, CA, USA).

## Results

### 3Demographic data

A total of 53 patients (106 hips) were included in the analysis (Table 1, Fig. 1). Among the 106 hips included in this study, all cases were secondary to developmental dysplasia of the hip (DDH), with 68 hips were classified as Crowe type I, 24 as type II, 13 as type III, and 1 as type IV (Table 2). The mean age of the patients was 63.6 ± 12.0 years. The mean height, body weight, and BMI were 152.2 ± 21.5 cm, 59.6 ± 12.7 kg, and 24.7 ± 5.1, respectively. Regarding spinopelvic alignment, the mean sagittal vertical axis (SVA) was 50.9 ± 45.0 mm, sacral slope (SS) was 39.1 ± 11.2°, and lumbar lordosis (LL) was 49.2 ± 14.7°. The mean preoperative mHHS was 52.5 ± 16.9, indicating moderate preoperative hip function.

**Table 1:** Demographic data

Demographic data	Mean (SD)
Age	63.6 (12.0)
Height	152.2 (21.5)
Body weight	59.6 (12.7)
BMI	24.7 (5.1)
Sagittal Vertical Axis	50.9 (45.0)
Lumbar Lordosis	49.2 (14.7)
Sacral Slope	39.1 (11.2)
Pre-Harris Hip Score	52.5 (16.9)

**Table 2:** Pearson’s correlation coefficients (r) between TotalSegmentator and manual segmentation across different degrees of morphological deformity according to the Crowe classification

		**Gluteus maximus**	**Gluteus medius**	**Paraspinal muscle**
Crowe type I (*N* = 68)	Volume	0.88	0.87	0.83
	CT attenuation	0.88	0.95	0.80
Crowe type II (*N* = 24)	Volume	0.94	0.90	0.89
	CT attenuation	0.91	0.95	0.90
Crowe type III, IV (*N* = 13, 1)	Volume	0.99	0.74	0.89
	CT attenuation	0.88	0.69	0.89

#### Validation of TotalSegmentator accuracy

**Fig. 3:**
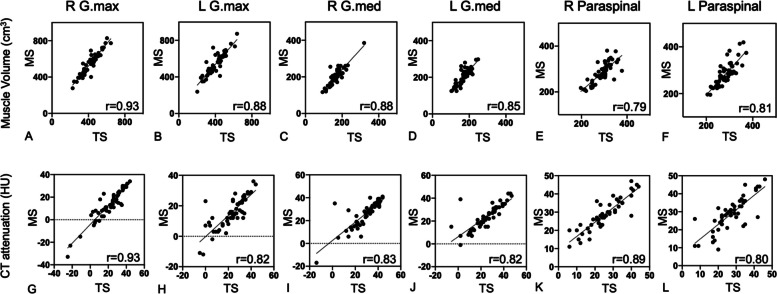
Agreement between automated and manual muscle measurements. Scatter plots illustrating the agreement between automated TotalSegmentator–based segmentation (TS) and manual segmentation (MS) for peri-hip muscle volume (cm^3^) and computed tomography (CT) attenuation values [Hounsfield unit (HU)]. Correlation coefficients (r) indicate excellent agreement between the two methods for both muscle volume and CT attenuation measurements across all evaluated muscle groups (**A**–**L**). Each point represents a single hip. Solid lines indicate the line of identity. (G.max: gluteus maximus; G.med: gluteus medius; R: right; L: left)

Intraclass correlation coefficients (ICCs) between TS and MS demonstrated moderate to good reliability for muscle volume measurements (ranging from 0.47 to 0.78) and good to excellent reliability for CT attenuation values (ranging from 0.67 to 0.83) (Table 3). These results indicate that TotalSegmentator provides quantitative measurements that are largely consistent with manual segmentation, particularly for paraspinal muscle evaluation. The correlation coefficients (r) between the TS and MS groups for the left and right gluteus maximus, gluteus medius, and paraspinal muscles were 0.93, 0.88, 0.88, 0.85, 0.79, and 0.81, respectively (Fig. 3A–F).

**Table 3:** Mean values and intraclass correlation coefficients (ICC) of the gluteus maximus, gluteus medius, and paraspinal muscles measured by TotalSegmentator and manual segmentation

**Parameter**		**Gluteus maximus**		**Gluteus medius**		**Paraspinal muscle**	
		**Right**	**Left**	**Right**	**Left**	**Right**	**Left**
Volume [(cm^3^), Mean (SD)]	TS	421.4 (94.2)	410.8 (93.5)	168.2 (36.7)	167.8 (34.4)	289.4 (40.1)	281.6 (44.7)
	MS	546.3 (122.7)	548.4 (120.6)	202.8 (47.6)	201.1 (45.8)	284.4 (37.8)	284.5 (50.4)
ICC Between TS and MS for Muscle Volume		0.541	0.470	0.618	0.601	0.779	0.784
CT attenuation [(HU), Mean (SD)]	TS	21.5 (15.2)	22.7 (12.3)	28.9 (12.0)	25.1 (12.3)	25.3 (9.5)	27.1 (8.9)
	MS	13.4 (13.7)	15.4 (10.3)	25.6 (11.6)	24.0 (10.6)	28.7 (8.3)	28.8 (9.0)
ICC Between TS and MS for CT attenuation		0.808	0.671	0.806	0.808	0.825	0.788

*TS* Total Segmentator, *MS* Manual Segmentation

**Fig. 4:**
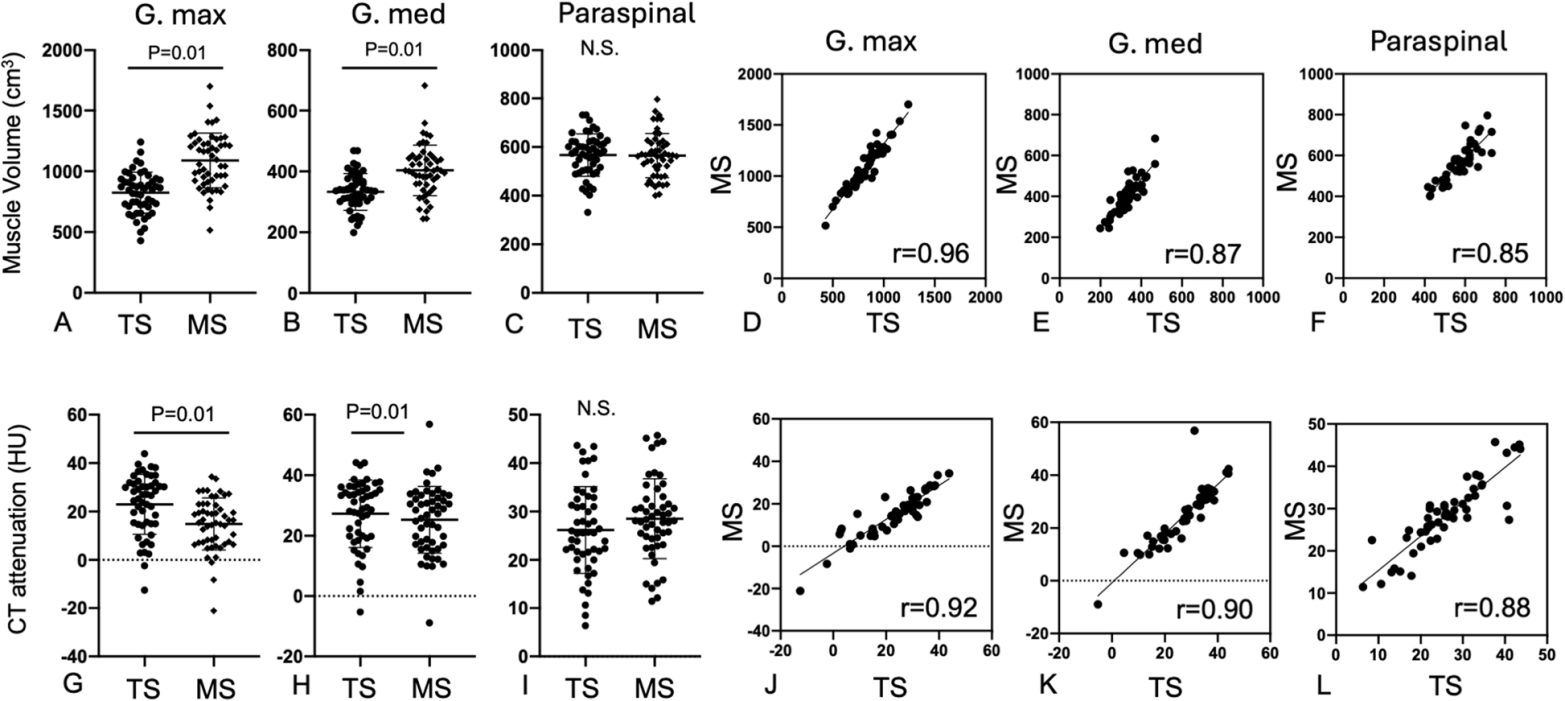
Differences and reproducibility between TotalSegmentator and manual muscle measurements. **A**–**C**, **G**–**I** Scatter plots comparing peri-hip muscle volume and computed tomography (CT) attenuation values obtained using automated TotalSegmentator-based segmentation (TS) and manual segmentation (MS), with measurements pooled from both the right and left hips. TotalSegmentator-based measurements yielded significantly lower muscle volumes and higher CT attenuation values in the gluteus maximus (G.max) and gluteus medius (G.med). *P* < 0.05 was considered statistically significant based on paired t tests. **D**–**F**, **J**–**L** Scatter plots demonstrating reproducibility for peri-hip muscle volume and CT attenuation values with measurements pooled from both hips. Solid lines indicate the line of identity. Correlation coefficients (r) indicate excellent reproducibility for both segmentation methods across all evaluated muscle groups

Similarly, CT attenuation values for these muscle groups also demonstrated strong correlations between the two methods, with r-values of 0.93, 0.82, 0.83, 0.82, 0.89, and 0.80, respectively (Fig. 3G–L). These results indicate that TotalSegmentator achieved consistent quantitative performance with manual segmentation. The mean (SD) muscle volumes of the gluteus maximus, gluteus medius, and paraspinal muscles were 421.4 (94.2), 168.2 (36.7), and 289.4 (40.1) cm^3^, respectively, on the right side, and 410.8 (93.5), 167.8 (34.4), and 281.6 (44.7) cm^3^, respectively, on the left side. The corresponding mean (SD) values measured by manual segmentation were 546.3 (122.7), 202.8 (47.6), and 284.4 (37.8) cm^3^ on the right, and 548.4 (120.6), 201.1 (45.8), and 284.5 (50.4) cm^3^ on the left (Table 3). When comparing the total volumes of the left and right gluteus maximus, gluteus medius, and paraspinal muscles, the TS group showed significantly smaller values than the MS group for both the gluteus maximus and gluteus medius (Fig. 4A–C, Table 3). Despite these differences, a strong correlation was still observed between the two methods (*r* = 0.85–0.96; Fig. 4D–E).

The mean (SD) CT attenuation values of the gluteus maximus, gluteus medius, and paraspinal muscles were 21.5 (15.2), 28.9 (12.0), and 25.3 (9.5) HU, respectively, on the right side, and 22.7 (12.3), 25.1 (12.3), and 27.1 (8.9) HU, respectively, on the left side. The corresponding MS-derived values were 13.4 (13.7), 25.6 (11.6), and 28.7 (8.3) HU on the right, and 15.4 (10.3), 24.0 (10.6), and 28.8 (9.0) HU on the left, respectively. The mean CT attenuation values of the gluteus maximus and gluteus medius were significantly higher in the TS group than in the MS group (Fig. 4G–I), although a strong correlation remained between the two methods (*r* = 0.88–0.92; Fig. 4J–L).

To investigate whether morphological changes of the hip influence these measurements, correlation analyses stratified by Crowe classification were performed. For muscle volume, the correlation coefficients in Crowe type I were *r* = 0.88, 0.87, and 0.83 for the G.max, G.med, and paraspinal muscles, respectively (Table 2). In Crowe type II, the corresponding values were *r* = 0.94, 0.90, and 0.89, while in Crowe type III, they were *r* = 0.99, 0.74, and 0.89 (Table 2). Similarly, for CT attenuation values, the correlation coefficients were *r* = 0.88, 0.95, and 0.80 in Crowe type I, *r* = 0.91, 0.95, and 0.90 in Crowe type II, and *r* = 0.88, 0.69, and 0.89 in Crowe type III for the G.max, G.med, and paraspinal muscles, respectively (Table 2).

Detailed examination of the segmentation patterns revealed that the MS group tended to include a broader area around the muscles, while the TS group showed more conservative segmentations that avoided including adjacent tissues. This systematic difference in segmentation boundaries is likely responsible for the statistically significant differences observed between the TS and MS groups.

The correlations among muscle volumes were *r* = 0.67 between the G.max and G.med, *r* = 0.54 between the G.max and paraspinal muscles, and *r* = 0.43 between the G.med and paraspinal muscles in the TS group. For CT attenuation, the correlations were *r* = 0.79 between the G.max and G.med, *r* = 0.45 between the G.max and paraspinal muscles, and *r* = 0.38 between the G.med and paraspinal muscles.

### Reproducibility of TotalSegmentator

To assess intraobserver reliability, TotalSegmentator was rerun after an interval of one month and evaluated for each target muscle. The intraclass correlation coefficients (ICCs) and Pearson’s correlation coefficients for the left and right gluteus maximus, gluteus medius, and paraspinal muscles were all 1.00, indicating perfect agreement in both muscle volume and CT attenuation values. These findings confirm the excellent reproducibility and consistency of TotalSegmentator across different computer environments.

### Associations between muscle parameters and patient characteristics or postoperative function

Exploratory secondary analyses were performed to evaluate associations between patient demographics, preoperative spinopelvic alignment, and pre- and postoperative hip functions and muscle parameters obtained by TotalSegmentator in 3D Slicer (Table 4). These analyses were not intended to validate segmentation accuracy itself.

**Table 4:** Correlations between patient background, spinopelvic alignment, and postoperative Harris hip score (HHS) with muscle evaluations

	**Muscle Volume (cm**^**3**^**)**			**CT attenuation (Hounsfield unit)**		
	**G.max**	**G.med**	**paraspinal**	**G.max**	**G.med**	**paraspinal**
Age	− 0.05	0.02	− 0.01	− 0.31*	− 0.33*	− 0.49*
Sagittal Vertical Axis	− 0.14	− 0.1	− 0.07	− 0.36*	− 0.39*	− 0.39*
Lumbar Lordosis	− 0.07	− 0.09	0.01	0.21*	0.18	0.40*
Sacral Slope	− 0.08	0.12	0.03	0.11	0.06	0.29*
Post-Op HHS	0.06	0.03	0.18*	0.16	0.18*	0.18*

G.max: gluteus maximus, G.med: gluteus medius; *Indicates *p* < 0.05 in Pearson’s correlation test

Higher CT density values of the G.max, G.med, and paraspinal muscles tended to be positively correlated with younger age, lower SVA, and higher LL and SS, although muscle volume showed no clear associations with these parameters (Table 4). The CT density of G.med and paraspinal muscles demonstrated a weak positive correlation with postoperative HHS.

## Discussion

Both 3D Slicer and its add-in software, TotalSegmentator, used in this study, are open-source platforms that are freely available to all users, which represents a major advantage in terms of accessibility and scalability [8, 10, 11]. Since muscle evaluation can be performed using CT data alone, this technique holds promise for screening and rehabilitation planning in patients suspected of having sarcopenia [13, 14] or locomotive syndrome [15, 16]. However, the training data for the automatic segmentation algorithm of TotalSegmentator were derived from CT scans of healthy individuals. Therefore, its accuracy in patients with musculoskeletal abnormalities, such as those with osteoarthritis, had not been previously validated. In the present study, all patients had bilateral hip osteoarthritis secondary to DDH, and many cases exhibited relatively severe deformity. Nevertheless, TotalSegmentator demonstrated a strong correlation with the manual segmentation results, indicating that 3D Slicer–based automated segmentation can reliably quantify muscle volume and CT density and may achieve segmentation performance comparable to that of orthopaedic surgeons, even in patients with anatomical deformity. This robustness was further supported by the stratified analysis according to Crowe classification, in which strong correlations were generally maintained even in advanced deformities (Crowe type III/IV), although a slightly lower correlation was observed for the G.med in these cases.

Manual segmentation of muscles from CT data typically requires 20 to 30 min per muscle; consequently, segmenting even six muscles can take 2 to 3 h per patient. In contrast, TotalSegmentator can segment all ten target muscles within approximately 5 min, representing a dramatic reduction in processing time. This improvement enables large-scale and objective muscle analysis to be performed more efficiently in both clinical and research settings. Reproducibility is an essential requirement for this rapid automated segmentation method. In the present study, even among patients with musculoskeletal disorders such as osteoarthritis, TotalSegmentator demonstrated perfect agreement even when repeated analyses were performed at different time points, confirming its excellent reproducibility and consistency. This stability ensures that segmentation results are not affected by temporal variations or operator-dependent factors, thereby enhancing the reliability of the method for longitudinal assessments and multicenter analyses.

Although TotalSegmentator demonstrated strong correlations and high reliability with manual segmentation, systematic differences in muscle volume and CT attenuation values were observed between the two methods. These discrepancies are likely attributable to differences in segmentation boundaries and muscle definition algorithms. TotalSegmentator generally delineates muscle regions more conservatively, often excluding peripheral areas with low attenuation or partial-volume effects. Consequently, in osteoarthritis patients with gluteal muscle atrophy, the segmented regions by TotalSegmentator tend to be smaller than those obtained by manual segmentation, resulting in significantly lower measured muscle volumes for the gluteus maximus and gluteus medius in our study. In contrast, manual segmentation tends to include broader peripheral areas of the muscle, which are more likely to exhibit fatty degeneration. Consequently, these marginal regions often show reduced CT attenuation, and their inclusion likely resulted in lower mean HU values in the MS group compared with the TS group. Importantly, this difference in HU does not indicate a segmentation error but rather reflects a fundamental difference in how each method defines muscle boundaries, with TotalSegmentator favoring geometric precision and manual segmentation emphasizing anatomical inclusion, especially in the gluteus muscles. In contrast, no significant difference was observed for the paraspinal muscles. This may be due to their more compact morphology and clearer fascial borders, which reduce the potential for boundary variability between the two segmentation methods. Additionally, in the present cohort, there were no cases in which the CT attenuation values of the paraspinal muscles fell below 0 HU, which may also have contributed to this finding. These findings suggest that while TotalSegmentator provides consistent and reproducible quantitative measurements, its conservative segmentation approach may underestimate muscle volume in atrophic conditions of gluteus muscles, yet offer reliable and reproducible CT attenuation estimates in patients with osteoarthritis.

With the rapid advancement of AI-based segmentation technologies, the availability of clinically relevant quantitative data in orthopedic practice is expected to expand substantially [17]. These developments may offer potential opportunities for integration into clinical decision-making, postoperative functional assessment, and personalized rehabilitation planning. However, further accumulation of muscle data and validation in diverse patient populations will be necessary to better clarify their clinical applicability. In our cohort, CT-based quantitative muscle measurements were weakly correlated with age, patient posture, and postoperative hip function. These results suggest that sarcopenia of the periarticular hip muscles in patients undergoing THA may have some relevance as an indicator of both baseline functional status and postoperative recovery potential, although the strength of these associations was limited. The present approach enables detailed quantification of individual muscle groups and may help to identify muscles that contribute most prominently to functional decline in each patient. With increasing case numbers and the establishment of age-stratified reference ranges, it may become possible to identify relatively vulnerable muscle groups preoperatively, which could potentially support more targeted and individualized rehabilitation strategies.

Previous studies have demonstrated that both muscle volume and CT attenuation correlate with muscle strength in THA patients and are associated with postoperative instability in revision settings [6, 18]. Because CT attenuation reflects tissue radiodensity, lower values indicate fatty infiltration or muscle fiber degeneration, consistent with muscle atrophy [19]. In the present study, paraspinal muscles showed weak correlations with preoperative SVA, LL, and SS, suggesting a possible effect on global spinal alignment. This finding highlights the potential of quantitative muscle analysis for paraspinal muscles to develop the rehabilitation programs aimed at improving posture, gait, ADL, and overall QOL in THA [20]. CT imaging with pelvic and femoral segmentation is already widely used in THA for preoperative planning, navigation, and robotic-assisted procedures [21–24]. Leveraging these existing CT datasets, TotalSegmentator enables automated assessment of peri-hip and paraspinal muscles within minutes, potentially enhancing preoperative risk stratification and postoperative outcome prediction. Moreover, similar applications may extend to traumatic conditions such as hip fractures, in which preoperative muscle weakness is common and quantitative muscle assessment may help predict postoperative ambulation and guide patient-specific rehabilitation strategies [25–30].

This study has several limitations. First, in its current form, TotalSegmentator does not enable quantitative evaluation of all peri-hip or lower extremity muscles, such as the short external rotators. The present analysis focused on three major muscle groups representing the core musculature involved in hip and spinopelvic function, rather than the entire peri-hip musculature. Consequently, its applicability in patients with musculoskeletal disorders is currently limited to the assessment of major periarticular hip muscles. Although we demonstrated that quantitative muscle assessment using CT data and TotalSegmentator can be performed easily and accurately, even in patients with severe deformities such as hip osteoarthritis, only selected muscle groups were evaluated. In addition, because segmentation was performed using CT data acquired in the supine position, the measurements reflect resting muscle morphology rather than weight-bearing or functional conditions. Nevertheless, given the observed correlations between muscle volume and CT attenuation, assessment of representative muscle groups may still provide useful insight into overall muscle degeneration. Second, all participants in this study were female patients with DDH. Because muscle volume, muscle composition, and the degree of fatty infiltration are known to differ substantially between sexes, the present findings should be interpreted as applying only to women with DDH. Therefore, the generalizability of our results to male patients or to individuals with other hip disorders remains uncertain. Finally, muscle parameters showed only weak correlations with postoperative hip function as assessed by mHHS. This may be partly attributable to the ceiling effect of the mHHS and its emphasis on pain-related outcomes rather than pure functional capacity. Moreover, postoperative ADL and QOL are influenced by multiple factors beyond peri-hip musculature, including spinal alignment, global sagittal balance, and systemic comorbidities, which may have confounded these associations. Future studies incorporating larger and more diverse cohorts, including male patients and individuals with various musculoskeletal disorders, as well as function-oriented outcome measures, are warranted to further refine and validate the algorithm, improve its generalizability, and better define the clinical utility of CT-based muscle assessment in guiding patient-specific rehabilitation strategies.

## Conclusion

Automated peri-hip muscle segmentation using TotalSegmentator provides accurate and highly reproducible quantitative assessment in patients with hip osteoarthritis. Although muscle volumes were smaller and CT attenuation values were higher than those obtained by manual segmentation, the overall agreement between methods suggests that this automated approach may provide reasonably reliable muscle assessment. TotalSegmentator could be a useful tool for rapid muscle evaluation in patients with hip osteoarthritis.

## Data Availability

The datasets used and/or analysed during the current study are available from the corresponding author on reasonable request.
